# Nitric oxide, thyroglobulin, and calcitonin: unraveling the nature of thyroid nodules

**DOI:** 10.3389/fendo.2023.1241223

**Published:** 2023-09-28

**Authors:** Vladimir S. Samardzic, Mirjana T. Macvanin, Sonja S. Zafirovic, Milan M. Obradovic, Zoran M. Gluvic, Jasmina Grubin, Xin Gao, Magbubah Essack, Esma R. Isenovic

**Affiliations:** ^1^ Clinic for Internal Medicine, Department of Endocrinology and Diabetes, Zemun Clinical Hospital, School of Medicine, University of Belgrade, Belgrade, Serbia; ^2^ Department of Radiobiology and Molecular Genetics, VINČA Institute of Nuclear Sciences - National Institute of the Republic of Serbia, University of Belgrade, Belgrade, Serbia; ^3^ Ministry of Science, Technological Development and Innovation of the Republic of Serbia, Belgrade, Serbia; ^4^ Computational Bioscience Research Center (CBRC), King Abdullah University of Science and Technology (KAUST), Thuwal, Saudi Arabia; ^5^ Computer Science Program, Computer, Electrical and Mathematical Sciences and Engineering Division, King Abdullah University of Science and Technology (KAUST), Thuwal, Saudi Arabia

**Keywords:** thyroid nodules, fine needle aspiration biopsy, nitric oxide, thyroglobulin, calcitonin

## Abstract

**Background:**

Thyroid nodules (TN) are localized morphological changes in the thyroid gland and can be benign or malignant.

**Objective:**

The present study investigates the relationships between biochemical markers in serum (s) and their homologs in washout (w) after fine-needle aspiration biopsy (FNAB) of the TN of interest and their correlation with cytology specimen findings.

**Methods:**

We investigated the relationships between serum biochemical markers nitric oxide (NO), thyroglobulin (TG), and calcitonin (CT), their homologs in washout after FNAB of the TN of interest, and cytology findings of biopsy samples classified according to the Bethesda system for thyroid cytopathology in this study, which included 86 subjects.

**Results:**

Washout TG (TGw) level positively correlates with the cytology finding of the biopsy. A higher level of TGw correlates with higher categories of the Bethesda classification and indicates a higher malignant potential. The levels of serum NO (NOs), serum TG (TGs), serum CT (CTs), and washout CT (CTw) do not correlate with the cytology finding of the biopsy, and the higher levels of washout NO (NOw) correspond to the more suspicious ultrasound findings.

**Conclusion:**

The findings of our study suggest that TGw and NOw could be used as potential predictors of malignancy in TN.

## 1 Introduction

Thyroid nodules (TN) are localized morphological changes in the form of lumps that occur in the thyroid gland (1), and the nodules can be benign or malignant (2, 3). It is estimated that the frequency of identifying palpable TN by physical examination is 2-6%, while TNs observed by ultrasound (US) are described in 19 to 49% of cases (2, 4, 5) and in 8-65% of autopsy cases (6, 7). Moreover, the incidence of malignant TN is significantly lower and accounts for <5% of all TNs (3). However, the number of TN and differentiated thyroid carcinoma (DTC) cases has increased recently (2, 8). TNs occur more frequently in the elderly, females, overweight individuals, smokers, and persons with insufficient iodine intake (5, 9).

US thyroid gland and neck lymph nodes (LN) examinations are recommended for all patients with known or suspected TN (1). Through the US, the characteristics of TNs can be described, such as their structure, echogenicity, size, shape, description of edges, calcifications, and vascularization (1). Since US diagnostics is highly subjective, systems for reporting and stratification of TN malignancy risk have been established to present US findings and allow their comparisons as uniformly as possible. The most commonly used systems for reporting changes in the thyroid gland visualized by the US are those recommended by the American College of Radiology Thyroid Imaging Reporting and Data System (ACR-TIRADS) and the European Thyroid Imaging Reporting and Association Data System (EU-TIRADS) (10, 11). Classifying an individual TN into a particular TIRADS classification further directs its diagnostic management (10).

Given the high frequency of TN with a relatively small percentage of malignant changes (1-5% of TNs), of particular interest is making a clear distinction between benign and malignant lesions to reduce the number of unnecessary surgical procedures, specifically, fine needle aspiration biopsy (FNAB) is performed. FNAB is an outpatient procedure using a thin needle (22 to 27G) under US control. The obtained material is used to make smears on glass slides, stained by the proper methods (12, 13). To interpret the cytology findings as uniformly as possible, the reporting system on thyroid cytopathology- Bethesda - was introduced in 2007, with the last revision in 2017 (14). Classification of TNs into one of the six groups of the Bethesda classification system carries a predefined risk of malignancy on which further diagnostic and therapeutic guidelines are based (14, 15). Cytology findings classified in groups Bethesda 4, 5, and 6 require histological verification of TNs (16, 17). US-guided FNAB has been accepted as the “gold standard” in the diagnostics of TNs (1). Nonetheless, non-diagnostic, i.e., undetermined cytology findings, are obtained in 15% -30% of biopsied TNs, while less than a third of malignancy is verified after histological analysis (18). One way to distinguish benign from malignant TNs is to determine the level of circulating biomarkers. Biomarkers, such as thyroglobulin (TG), anti-thyroglobulin antibodies (ATGAbs), and calcitonin (CT), are routinely used in preoperative diagnosis and postoperative follow-up of patients with TN, DTC, and medullary thyroid cancer (MTC) (19, 20). In addition, there is an intensive ongoing evaluation of the clinical utility of circulating biomarkers such as mRNA and microRNA (21, 22).

Taking into account the facts regarding the complexity of the TN diagnostic procedures, the present study investigates the relationships between biochemical markers in serum (s) and their homologs in washout (w) after FNAB of the TN of interest, and cytology findings of biopsy sample classified according to the Bethesda system for thyroid cytopathology, with the aim of improved and more accurate identification of the TN of interest. Therefore, we measured the nitric oxide (NO), TG, and CT levels in the washout of TN after FNAB (NOw, TGw, CTw) and in the serum of the patients (NOs, TGs, CTs). In addition, we also analyzed the relationships between cytology findings of the FNAB and the level of NO, TG, and CT in the TN washout and the subjects’ serum.

Our preliminary results indicate higher levels of NO in FNAB washouts in patients with malignant TN compared to benign TN (23–25), and the present study with a larger cohort is performed to elucidate further oncogenic NO potential, presumably in malignant TN.

NO has equivocal roles in malignancies. It impacts either tumorigenesis initiation or prevention (25). NO can activate and stabilize p53, hypoxia-inducible factor (HIF), Akt and ERK (26). NO also contributes to matrix remodeling, neovascularization, tumor growth, and local and distant thyroid malignancy spreading by affecting tissue inhibitors of metalloproteinase (TIMP), matrix metallopeptidases (MMP), vascular endothelial growth factor D (VEGF-D), and C-X-C chemokine receptor type 4 (CXCR4) (26–28). Furthermore, NO could directly or indirectly influence DNA methylation and histone modification, consequent inactivating tumor suppressor genes (29). Additionally, the end-products of NO damage DNA and repair mechanisms at replication and posttranslational levels and favor the apoptosis of healthy cells (30).

## 2 Materials and methods

### 2.1 Definition and selection of study participants

The cross-sectional study included 86 subjects who reported to the outpatient endocrinology examination at the University Clinical Hospital Zemun (UCHZ) from January 2019 to November 2019. The Ethics Committee of UCHZ approved the study (approval number 507/1), and it was conducted following the code of ethics of the World Medical Association (Declaration of Helsinki), printed in the British Medical Journal (July 18, 1964).

The primary study inclusion criterion was the presence of TN > 1cm in the largest diameter detected by the US, with the US finding corresponding to groups 3, 4, and 5 according to the EU-TIRADS classification (10). Subjects who met the inclusion criterion signed written consent for participation in the study.

We performed a clinical examination after recording the gender and age of all subjects. The clinical examination included neck inspection and palpation, US examination of the thyroid gland and neck, and blood sampling to determine thyroid-stimulating hormone (TSH), TG, CT, and NO biochemical markers, followed by FNAB.

### 2.2 US examination of the thyroid gland

US examination of patients’ thyroid glands was performed by an examiner certified for US diagnostics. Examinations were performed on a Toshiba Xario SSA-660A US device with a 7.5MHz linear probe. Each TNs’ size was measured in three dimensions (longitudinal, sagittal, and transverse), after which the TNs of interest were classified according to the EU-TIRADS classification. Respondents with TN classified in EU-TIRADS 3, 4, and 5 met the basic inclusion criterion.

### 2.3 Blood sampling and isolation of serum

Subjects were given written instructions with advice to avoid consuming certain foods whose ingredients may affect the monitored biochemical parameters in the study after being selected for the study and recording their demographic parameters (sex and age). For example, the subjects were advised to avoid foods such as spinach, beets, and lettuce containing high nitrite (NO_2_¯) levels, affecting the validity of measured NO levels (31). All respondents were instructed to adhere to the regimen from 8 pm before the morning blood sampling.

Blood for NO, TG, CT, and TSH determination was sampled immediately before performing FNAB. The collected blood was incubated for 30 minutes at room temperature and then centrifuged for 15 minutes at 3500 rpm to isolate sera. Supernatants obtained after centrifugation were aliquoted into smaller volumes. The TSH, TG, and CT levels were determined 72 h after sampling, whereas the aliquots for NO measurements were frozen and stored at -20° C for further analysis.

### 2.4 Biopsy and sampling of TN washouts

FNAB was performed on all subjects following blood sampling, with aspiration of material from the TNs using a 26G needle, under US control by the same US device on which the diagnostic US was performed, and carried out by the same endocrinologist who had performed FNAB previously. Smears of the obtained material were made on glass slides and stained using the May-Grünwald-Giemsa method. A pathologist of UCHZ with experience in TN cytology subsequently evaluated the slides.

After smearing, the remaining material in the FNAB needle was washed with 1ml of physiological solution and aliquoted into smaller volumes for further biochemical analysis. TG and CT levels were determined from a portion of the wash samples within 24 h after sampling, while aliquots used to determine NO were frozen and stored at -20°C.

Cytology findings were classified according to the Bethesda classification system (14); subjects with cytology findings belonging to Bethesda 4, 5, and 6 were referred for surgical treatment (lobectomy or total thyroidectomy). Postoperative histological findings were defined as benign or malignant.

### 2.5 Measurements of levels of TSH, TG, and CT in sera and TN FNAB washouts

The biochemical parameters were determined in the biochemical laboratory of UCHZ. We determined TSH levels (mIU/ml) using a commercial test on an automatic Access-2 “Beckman Coulter” analyzer. The reference values ​​for TSH are 0.38-5.33 mIU/ml. TG levels were determined using commercial tests on the UniCel DxI 600 Beckman Coulter automated analyzer, while CT levels were determined using commercial tests on the Roche Hitachi Cobas e411 automated analyzer. For both sexes, reference values ​​for TGs are 1.59-50.30 ng/ml, and CTs are <11.5 pg/ml. We determined the TGw and CTw levels using the same analyzers as the serum homologs. TGw values ​​are expressed in ng/ml and CTw in pg/ml, with non-standardized reference values ​​for these analyses.

### 2.6 Measurements of NO levels in serum and TN washout

The NOs/w levels were determined indirectly by measuring NO_2_¯ and nitrate (NO_3_¯) levels as NO end products using a commercial kit (Nitrate/Nitrite Colorimetric Assay Kit, Cayman Chemical IN 780001) and following the manufacturer’s instructions. The NO_2_¯/NO_3_¯ levels were determined in two steps. In the first step, NO_3_¯ is converted to NO_2_¯ by adding the enzyme nitrate reductase. In the second step, NO_2_¯ is converted to the azo compound by adding the Griess reagent. The concentration of NO_2_¯ is determined by measuring the absorbance of the formed dark purple azo compound. The absorbance was read at 540 nm in an automatic microtiter plate reader (Perkin Elmer, Wallac 1420 Victor). The NO_2_¯ levels are expressed in μM. To normalize NOw with TGw as the mass equivalent of the sampled thyroid tissue, we defined the TGw/NOw indices for each subject in the study.

### 2.7 Statistical methods

Statistical methods used in this study were methods of descriptive and analytical statistics. Methods of descriptive statistics included relative numbers, measures of central tendency, and measures of variability. Arithmetic means, and median were used as measures of central tendency, and standard deviation, interval, and coefficient of variation were used as measures of variability. Analytical statistics included tests for assessing the significance of correlations and differences. Spearman’s rank correlation test was used to determine the correlation’s significance. To determine the significance of the difference, the t-test for independent samples was used in the case of parametric data, and the χ^2^ test in the case of categorical, i.e., the Mann-Whitney test in cases of interval data that do not follow the normal distribution. In cases where the significance of the difference between the three groups of subjects was examined, one-factor parametric and nonparametric analyses of variance (ANOVA and Kruskal-Wallis) were used. The level of statistical significance is 0.05. The obtained data were analyzed using the statistical package SPSS for Windows 18.0.

## 3 Results

### 3.1 EU-TIRADS classification

The study included 86 subjects with an average age of 60 ± 12 (33–79) years, which did not differ between groups of subjects (F = 1.169; DF = 2; p> 0.05). Of the total number of subjects, 76 (88%) are female. The distribution of frequencies of patients according to gender participation differed significantly between groups (χ^2 = ^6.016; DF = 2; p <0.05). No statistically significant influence of gender participation (ρ = 0.102, p> 0.05) and age of respondents (ρ = -0.050, p> 0.05) on the categories of the EU-TIRADS classification was observed.

The median TN diameter is 21 (10–72) mm, while the mean serum TSH level is 1.39 ± 1.03 (0.00-5.73) mIU/ml. Mean TSH levels did not differ between EU-TIRADS groups. The association of TSH levels with EU-TIRADS classification categories was detected but did not reach statistical significance (ρ = 0.200, p = 0.07). Regarding the EU-TIRADS classification of TN of interest, 46% (32), 49% (33), and 4 (4) of subjects belonged to the EU-TIRADS categories 3, 4, and 5, respectively.

### 3.2 Biochemical markers in sera and TN biopsy washout

Mean levels of biochemical markers in sera and TN biopsy washouts are given in **Table 1**, while the frequency distribution of subjects by Bethesda classification categories is shown in **Table 2**. A total of 20 (23%) subjects were surgically treated.

**Table 1:** Median levels of biochemical markers measured in serum and TN washouts.

Biochemical marker	S	W
TG [Med (min-max)]	34.0 (0.1-496.0)	11.5 (0.0-99.7)
CT [Med (min-max)]	2.0 (2.0-46.6)	2.0 (2.0-3.9)
NO [sX ± SD/w Med (min-max)]	35.1 ± 19.8 (7.9-90.8)	3.4 (0.0-24.5)

CT, calcitonin; NO, nitric oxide; S, serum; TG, thyroglobulin; TN, thyroid nodules; W, washout.

**Table 2:** Distribution of patients according to the categories of Bethesda classification.

Bethesda category	n	%
1	19	22
2	40	47
3	8	9
4	14	16
5	4	5
6	1	1
Σ	86	100

Σ-sum, n-number of patients.

**Figure 1:**
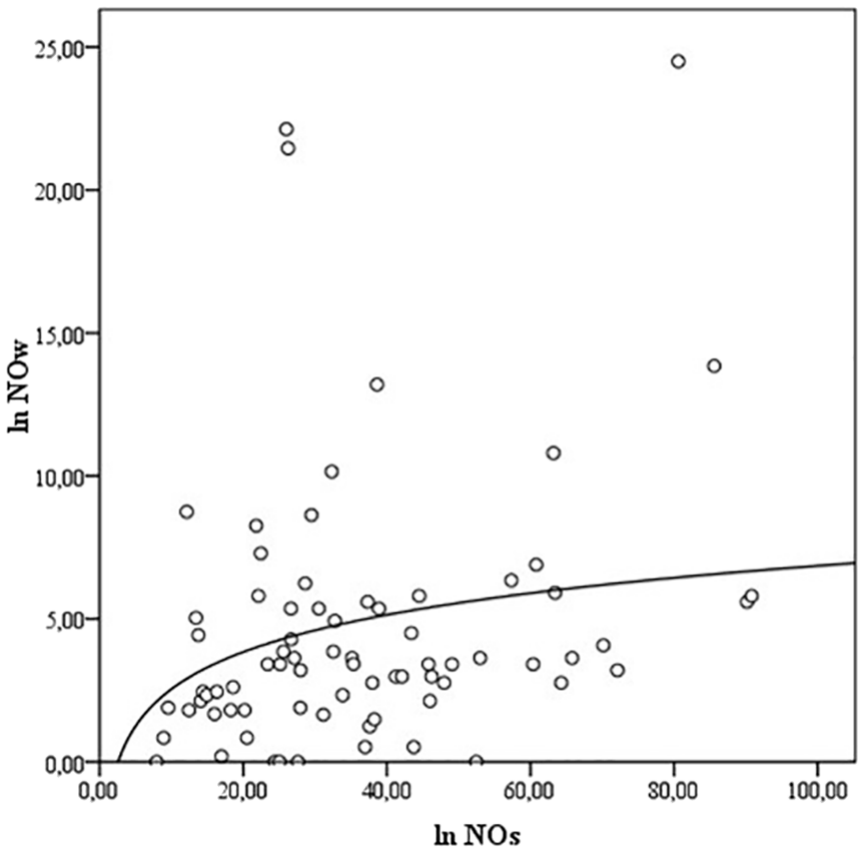
The correlation between the levels of NOs and NOw. NO- nitric oxide, NOs, serum NO; NOw, washout NO.

**Figure 2:**
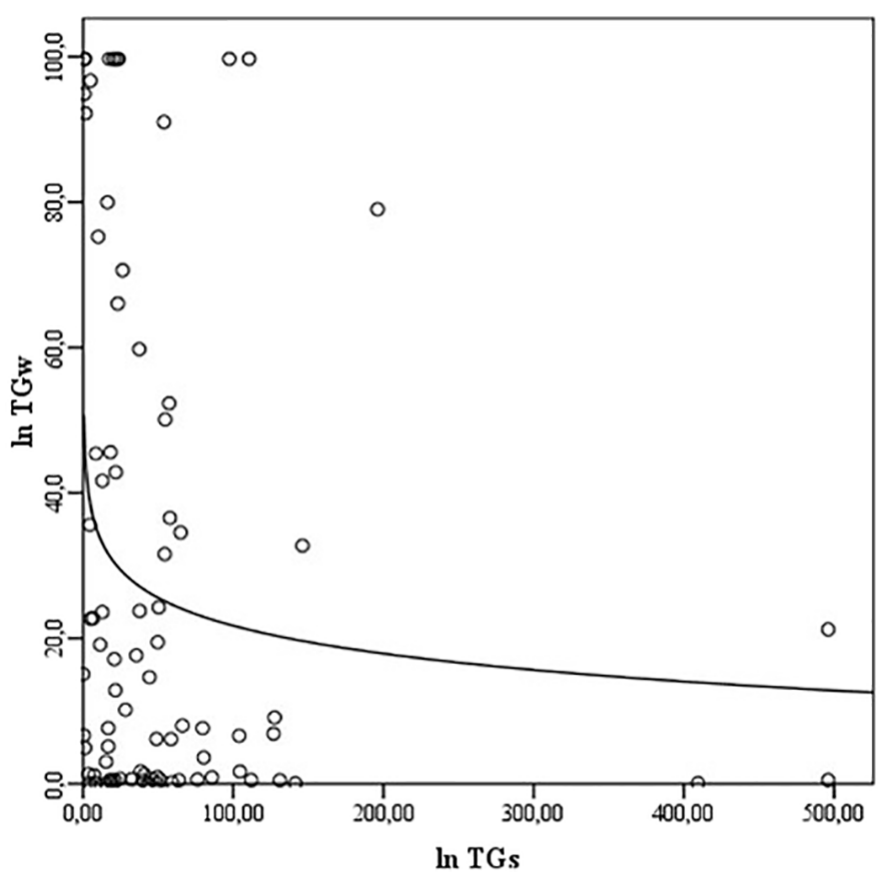
The correlation between the levels of TGs and TGw. TG, thyroglobulin; TGs, serum TG; TGw, washout TG.

NOs and NOw levels correlate with each other (ρ = 0.289, p<0.01) (**Figure 1**), but no association of NOs and NOw levels with Bethesda classification categories was observed (ρNOs/B = -0.028; ρNOw/B = 0.097, p>0.05). Also, there is a correlation between TGs and TGw levels (ρ = -0.208, p<0.05) (**Figure 2**). However, while the levels of TGw positively correlated with the categories according to the Bethesda classification (ρ = 0.250, p <0.05), no correlation was observed between the levels of TGs and the Bethesda categories (ρ = 0.041, p>0.05).

There is no correlation between the levels of CTs and CTw (ρ = -0.100, p>0.05), nor do they affect categories according to the Bethesda classification (ρCTs/B = 0.082; ρCTw/B = 0.060, p>0.05).

The mean NOw/TGw ratio in the study population was 0.25 (0-72.81). This ratio correlates with the levels of TGs (ρ = 0.235, p <0.05), TGw (ρ = -0.750, p<0.01), NOs (ρ = 0.385, p <0.01) and NOw (ρ = 0.417, p<0.01). No correlation of NOw/TGw ratio with CTs (ρ = 0.126, p> 0.05) and CTw (ρ = -0.086, p>0.05) was observed. Also, the NOw/TGw ratio did not correlate with the EU-TIRADS categories (ρ = 0.07, p>0.05) or Bethesda classification (ρ = -0.062, p>0.05), nor with the findings of postoperative histology (ρ = -0.010, p>0.05).

The positive correlation between EU-TIRADS classification and the Bethesda classification (ρ = 0.179, p=0.09), and EU-TIRADS classification and histology findings in surgically treated subjects (ρ = 0.400, p=0.08) did not reach statistical significance. Except for NOw levels (ρ = 0.231, p<0.05), EU-TIRADS classification categories do not correlate with other monitored biochemical markers in serum and TN biopsy washouts.

Analysis of data from the population of surgically treated subjects (n = 20) shows that histological findings confirming the malignant nature of TN were registered in 5 (25%) subjects. Groups of subjects based on histological findings differ statistically in the distribution of frequencies of subjects by categories of EU-TIRADS classification (χ^2 = ^6,800; DF = 2; p<0.05), whereas this difference is not registered relative to sex, age, TSH levels, category Bethesda classification, as well as to monitored biochemical parameters in serum and TN biopsy washouts. **Tables 3**, **4** show data by groups of subjects, according to the EU-TIRADS and Bethesda classification.

**Table 3:** Presentation of clinical, biochemical and histological data of patients in relation to the EU-TIRADS classification.

Variable		EU-TIRADS 3 (n=40)	EU-TIRADS 4 (n=42)	EU-TIRADS 5 (n=4)	p
*Sex [♀(%)]*		36 (90)	38 (90.5)	2 (50)	**<0.05**
*Age* *[X ± SD (min–max)]*		59 ± 12 (36-78)	60 ± 11 (36-79)	50 ± 15 (33-68)	ns
*Diameter of nodule* *[Med min–max)]*		25 (13-72)	19.5 (10-35)	14.5 (13-45)	**<0.01**
*TGs* *[Med (min–max)]*		34.9 (0.6-496.0)	36.9 (0.1-196.1)	14.0 (4.5-49.8)	ns
*TGw* *[Med (min–max)]*		15.9 (0.0-99.7)	7.6 (0.0-99.7)	27.5 (0.0-45.4)	ns
*CTs* *[Med (min–max)]*		2.0 (2.0-46.6)	2.0 (2.0-8.2)	2.6 (2.0-6.8)	ns
*CTw* *[Med (min–max)]*		2.0 (2.0-3.6)	2.0 (2.0-3.9)	2.0 (2.0-2.8)	ns (0.07)
*NOs* *[X ± SD (min–max)]*		34.6 ± 20.9 (8.9-90.8)	35.2 ± 19.2 (7.9-85.6)	38.3 ± 18.0 (22.4-63.4)	ns
*NOw* *[Med (min–max)]*		2.9 (0-22.1)	3.7 (0-24.5)	6.8 (2.5-13.2)	ns (0.07)
*NOw/TGw* *[Med (min–max)]*		0.20 (0-72.81)	0.32 (0-70.76)	0.44 (0.13-72.77)	ns
*TSH* *[X ± SD (min–max)]*		1.29 ± 1.22 (0-5.73)	1.45 ± 0.79 (0-3.51)	1.80 ± 1.29 (0.89-3.66)	ns
*Bethesda* *category* *n (%)*	*1*	9 (22.5)	10 (24)	0	ns
	*2*	22 (55)	17(40.5)	1 (25)	
	*3*	3 (7.5)	4 (9.5)	1 (25)	
	*4*	6 (15)	7 (17)	1 (25)	
	*5*	0	3 (7)	1 (25)	
	*6*	0	1 (2)	0	
*Surgery [(%)]*		8 (40)	10 (50)	2 (10)	ns
*Histology finding* *[n (%)]*	*benign*	7 (88)	8 (80)	0	**<0.05**
	*malignant*	1 (12)	2 (20)	2 (100)	

CT, calcitonin; EU-TIRADS, European Thyroid Imaging and Reporting Association Data System; n, number of patients; NO, nitric oxide; ns, non-significant; s, serum; TG, thyroglobulin; TSH, thyroid stimulating hormone; w, washout. The bold emphasizes statistically significant difference.

**Table 4:** Presentation of clinical, biochemical and histological data of patients with the Bethesda classification.

Variable		B1 (n=19)	B2 (n=40)	B3 (n=8)	B4 (n=14)	B5 (n=4)	B6 (n=1)
*Sex [♀(%)]*		17 (89.5)	36 (90)	8 (100)	11 (78.5)	3 (75)	1 (100)
*Age* *[X ± SD (min–max)]*		56 ± 12 (38-76)	61 ± 11 (36-79)	55 ± 15 (36-77)	60 ± 13 (33-78)	58.5 ± 10 (45-69)	55
*Diametar of nodule* *[Med (min–max)]*		21 (13-34)	23 (13-72)	20 (10-39)	20 (17-45)	17.5 (14-23)	19
*TGs* *[Med (min–max)]*		38.4 (0.1-131.0)	31.9 (0.6-496.0)	42.1 (16.3-496.0)	34.8 (0.9-141.3)	22.4 (4.5-65.0)	126.8
*TGw* *[Med (min–max)]*		1.25 (0-42.8)	23.2 (0-99.7)	0.6 (0-99.7)	21.85 (0.1-99.7)	35.1 (0.7-99.7)	6.9^**^
*CTs* *[Med (min–max)]*		2.0 (2.0-17.4)	2.0 (2.0-8.2)	2.0 (2.0-46.6)	2.0 (2.0-6.5)	2.0 (2.0-5.0)	2.0
*CTw* *[Med (min–max)]*		2.0 (2.0-2.0)	2.0 (2.0-3.9)	2.0 (2.0-2.0)	2.0 (2.0-2.8)	2.0 (2.0-2.0)	2.0
*NOs* *[X ± SD (min-max)]*		31.9 ± 14.8 (7.9-63.2)	38.2 ± 22.5 (9.5-90.8)	30.3 ± 16.6 (12.4-63.4)	33.5 ± 21.0 (12.1-80.6)	36.5 ± 17.4 (21.8-57.3)	25.15
*NOw* *[Med (min–max)]*		2.9 (0-22.1)	3.6 (0.8-16.3)	2.9 (0-5.0)	3.5 (0.2-24.5)	5.5 (0.2-7.3)	3.41
*NOw/TGw* *[Med (min–max)]*		0.64 (0-72.81)	0.16 (0.008-70.76)	1.35 (0-72.78)	0.45 (0.019-48.91)	0.18 (0-7.31)	0.49
*TSH* *[X ± SD (min–max)*		1.63 ± 0.81 (0.45-3.51)	1.19 ± 0.98 (0-5.42)	1.69 ± 0.67 (0.95-2.53)	1.52 ± 1.44 (0.02-5.73)	1.70 ± 1.35 (0.61-3.66)	0.01
*Surgery* *[n (%)]*		3 (15)	0	3 (15)	11 (55)	2 (10)	1 (5)^**^
*Histology finding* *[n (%)]*	*benign*	3 (100)	0	2 (67)	9 (82)	1 (50)	0
	*malignant*	0	0	1 (33)	2 (18)	1 (50)	1 (100)

B, a group according to the Bethesda classification; CT, calcitoni; n, number of patients; NO, nitric oxide; ns, non-significant; s, serum; TG, thyroglobulin; TSH, thyroid stimulating hormone; w, washout; ^**^- p<0.01.

## 4 Discussion

### 4.1 The distribution of respondents into different EU-TIRADS groups

Ultrasound examination of the thyroid gland is the conventional diagnostic procedure for evaluating TNs (10). Moreover, US characteristics, such as nodule structure and shape, nodule echogenicity and margin to the surrounding tissue, vascularization of the nodule, and calcification, have been used to develop several classification systems. Classification of TNs into defined groups describes more uniformly the US findings based on individual characteristics, thus stratifying the risk of malignancy and directing further diagnostic management. Therefore, we divided the patients in our cross-sectional study into three groups (TIRADS 3, 4, and 5) according to the EU-TIRADS classification system recommended by the European Thyroid Association (10, 11). These three EU-TIRADS groups are of significant clinical interest because the subjects in these groups potentially have malignant TN. The more substantial number of patients investigated in our study (49%) belonged to groups 4 (49%) and 3 (46%), while only 5% were placed in group 5. The distribution of respondents into different EU-TIRADS groups in our study aligns with the previously published data (2, 5, 9). We observed a slightly higher ratio of female to male subjects (7.6:1) in our study group compared to data described in the literature (1, 34), which can be explained by a relatively small sample of patients recruited in only one clinical center.

### 4.2 Classification of the cytology findings

US-guided FNAB is the “gold standard” in the diagnostics of thyroid tumors (14), which facilitates standardization of the cytology findings to further stratify TN malignancies’ risk and aids in precise preoperative recruitment of patients. We used the Bethesda classification system (17, 35) to classify our cytology findings into six groups. Precisely, of the eighty-six subjects included in our study, nineteen subjects (22%) were placed in Bethesda 1 group (inadequate sample), and forty subjects (47%) in Bethesda 2 group (benign finding) (**Table 2**). The other twenty-two (25%) subjects were placed in classification groups with undetermined cytology, which included eight subjects (9%) in the Bethesda 3 group (atypia/follicular lesion of undetermined significance), fourteen subjects (16%) in the Bethesda 4 group (follicular neoplasm/suspicious of follicular neoplasm), and four (5%) subjects in the Bethesda 5 group (suspicious for malignancy) (**Table 2**). Only one subject was placed in the Bethesda 6 (malignant) group (**Table 2**). Because of non-conclusive cytology findings, we repeated FNAB for six out of eight subjects, with initial cytology findings classified in the Bethesda 3 group. Based on the repeated cytology analysis, four were placed in the Bethesda 2 group and two in the Bethesda 4 group. The other two subjects with an initial cytology finding of the Bethesda 3 group refused to repeat FNAB and the consequent surgical treatment and opted for periodic US monitoring. In our study group, we notice a slightly higher frequency of inadequate samples from the Bethesda 1 group, whereas the distribution in other groups follows the literature data (36, 37).

Although routine use of FNAB in preoperative evaluation of TN reduces unnecessary surgical interventions for histological examination of TN [7], literature data show that there is still a relatively high frequency of undetermined and non-conclusive cytology findings obtained in 15-30% of analyses of samples belonging to Bethesda groups 3 and 4. Since follicular thyroid carcinoma differs histologically from follicular adenoma through capsule invasion by tumor cells, cytology analysis of FNAB-derived samples cannot distinguish these two entities (14). Cytologically indeterminate TNs have a sufficiently high risk for malignancy (5-75%) and require further evaluation (16, 17). Surgical treatment is performed to obtain a definitive histological finding, and the consequent analysis of the combination of cytology results and the intraoperative and ex tempore biopsy findings influences the decision to perform a lobectomy or total thyroidectomy. If compressive symptoms are severe or TN is suspected in the presence of pathologically altered LN in the neck, patients without a history of FNAB or a benign cytology finding are also surgically treated (especially if microcalcifications or cystic LN degeneration are observed) (38, 39). Subjects with cytology findings in Bethesda groups 4, 5, and 6 were referred for surgical treatment in our study (lobectomy or total thyroidectomy). Twenty out of twenty-seven subjects (74%) who were advised for surgery were treated. Five patients refused the proposed surgical treatment, and the other two cases did not follow up for further monitoring.

Based on the postoperative TN histology verification, our results show that most TNs in patients with cytologically indeterminate findings are benign, aligning with other studies’ findings. For instance, a study that included 499 patients with TN (40) showed, based on postoperative histology findings, that 70% of patient TN’s categories in the Bethesda 3 group and 72.3% of patients TN’s categories in the Bethesda category 4 are benign. Another multicenter study that included 1501 FNAB samples from 1285 TNs (18) also showed that 66% of benign histology findings were from TNs with indeterminate cytology.

### 4.3 Correlation between TN cytology findings and the biochemical markers

To provide a more precise preoperative assessment of patients in the Bethesda 3 and 4 categories, two tests based on the “genetic profile” of the TN were developed to distinguish between benign and malignant changes (32, 41). The “*Rule in*” tests detects the existence of one or more of the most common genetic mutations and/or rearrangements found in malignant TN (BRAF mutations, point mutations of the RAS gene, fusion genes RET/PTC1, RET/PTC3, and PAX8/PPARγ) (33, 42). While the “*Rule out*” tests exclude TNs’ malignant nature based on the presence/absence of specific genetic mutations. Several commercial tests with relatively high sensitivity (~ 95%) and negative predictive value (~ 95%) are available (Afirma G.S.C., ThyroSeqv3 GC) (20, 43). However, due to the high cost of assembling and analyzing patients’ genetic profiles, it is still not widely accessible for routine use in the diagnostics of TNs in the clinical setting. Opportunely though, determining biochemical markers in FNA washout fluid is emerging as a powerful, accessible, and inexpensive alternative to genetic profiling that can reliably refine clinical diagnosis in patients with thyroid tumors (44). For example, it has been shown that the diagnostic accuracy of FNA analysis is significantly increased by measuring TG and CT levels in the FNA washout fluid (45), and these measurements are included in the current clinical guidelines (46).

#### 4.3.1 Thyroglobulin

TGs are a circulating tumor marker in follow-up DTC patients with total thyroidectomy (20, 47). Also, increased TGs values ​​in the postoperative period suggest recurrent or metastatic disease (1). However, since elevated TGs values ​​ are observed in various thyroid diseases (goitre, thyroiditis, benign and malignant nodules), the routine determination of TG levels in preoperative evaluation of patients with TN or screening of malignant diseases of the thyroid gland is not advised (48, 49). Nonetheless, several studies (19, 50) indicate the potential significance of preoperatively detected elevated TG levels in patients with follicular thyroid carcinoma and Huerthle cell carcinoma.

Also, although it is acknowledged that TGw levels after TN FNAB represent a diagnostic method for evaluating suspected metastatic LN in the neck (46), Jee et al. reported lower TGw levels in malignant relative to benign TN (51), which may be a consequence of the amount of thyroid tissue sampled. Thus, since the level of TGs is proportional to the mass of thyroid tissue, the principal reason for determining the levels of TGs in our study was to measure the amount of sampled thyroid tissue. This method allowed us to eliminate the effect of differences in thyroid tissue amount on NOw and TGw levels (51). Our results show TGs values did not correlate with cytology and histology findings in the surgically treated subjects (**Tables 3**, **4**). However, in contrast to TGs levels, we observed that TGw levels positively correlated with cytology findings.

#### 4.3.2 Calcitonin

CT is a circulating biomarker for detecting C cell hyperplasia (CCH) and MTC (52, 53). It is synthesized and secreted by parafollicular or C cells of the thyroid gland and MTC tumor cells. The concentration of CT is proportional to the mass of C cells, which is why the determination of CT has diagnostic significance both in the diagnosis of MTC and in the postoperative follow-up of patients with MTC (54). Routine determination of CT levels in evaluating patients with TN increased the detection of MTC in patients with TN, from 1.1 to 3.2 per 1000 TN. The increase in the incidence of MTC is primarily due to the increased detection of occult MTCs, but it is simultaneously accompanied by an increase in false-positive findings, which resulted in the exposure of patients to unnecessary surgical treatment. The value of basal CT with the highest sensitivity (89%) and specificity (75%) that discriminates between patients with CCH and those with MTC is 15 pg/ml for women and 80 pg/ml for men (55). A meta-analysis that included 15 studies evaluating the use of FNAB in MTC diagnostics showed that the method’s sensitivity in detecting MTC was <50% (45). Detecting MTC is significantly increased by immunohistochemical detection of CT, carcinoembryonic antigen (CEA), chromogranin A (CgA), and determining CTw levels after FNAB. Moreover, a multicenter study by Trimboli et al. (45) showed that measurements of CTw have significantly greater detection sensitivity than cytology findings.

Our study observed no correlation between the levels of CTs and CTw. Also, CTs and CTw did not correlate with cytology findings grouped according to the Bethesda classification or histology observed subjects operated on (**Table 4**). This result is expected, given the small sample size and no CCH or MTC was detected based on the histology findings.

#### 4.3.3 Nitric oxide

NO can exhibit anti-tumor or tumorigenic activity depending on its target tissue concentration (25). That is, studies show low levels of NO (<100nM) promote angiogenesis and proliferation, medium levels of NO (100-500nM) increase invasiveness, propensity for metastasis, and suppression of apoptosis, whereas high levels (> 500nM) lead to cytotoxicity, macromolecule degradation, and apoptosis (25, 56). The harmful effects of NO observed in malignancies are explained through several mechanisms. When present in excess, NO reacts with superoxide anions, leading to highly reactive peroxynitrites and other reactive nitrogen species. Peroxynitrite leads to DNA damage, suppression of DNA repair mechanisms, posttranslational enzyme modifications, and inhibition of apoptosis (57, 58). NOs’ harmful effects influence the tumor microenvironments’ redox status and the cell cycle phase (59). Patel et al. (59) showed increased expression of inducible nitric oxide synthase (iNOS), endothelial NOS (eNOS), and nitrotyrosine in the tissues of benign thyroid adenomas, PTC, Follicular thyroid carcinoma, and autoimmune thyroiditis. Yasuoka et al. (28) showed decreased expression of iNOS in normal thyroid follicular cells and an increased expression of iNOS in thyroid tumor cells, with poor immunoreactivity to iNOS in stromal, indicating that the majority of produced NO originates directly from tumor cells. Increased iNOS expression and NO concentration in PTC cell culture increased VEGF-D expression, resulting in angiogenesis and metastatic tumor potential (27). Furthermore, NO has been shown to increase the expression CXCR4 in PTC cases, leading to the development of lymphogenic metastases (60, 61). Increased iNOS expression and changes in NO levels are not only characteristic of malignancies of the thyroid gland but also various other malignancies associated with inflammation (62), such as lung cancer (63), colorectal cancer (64), breast cancer (65) pancreatic cancer (66) and laryngeal cancer and melanoma (67). Thus, it is plausible to expect elevated NO levels in the tissues affected by the malignant processes and in the serum of patients with malignancies.

Our findings show a positive correlation between NOs and NOw levels in benign and malignant TN patients. NO levels did not differ significantly among subjects classified based on cytology and histology findings, whereas higher NOw levels were observed in subjects classified as higher in the EU-TIRADS classification (**Table 3**). However, the difference between groups did not reach statistical significance (p=0.07). The NOw/TGw ratio increased in groups with a higher risk of malignancy according to the EU-TIRADS classification, but the difference did not reach statistical significance (p=0.07) (**Table 3**). Also, we observed no association between NOw/TGw and cytology or histology findings. The NOw/TGw ratio correlates with NOw/s and TGw/s but not CTw/s. A quarter of the total number of surgically treated subjects they had histologically verified malignant nature of nodular thyroid disease. Even though the number of surgically-treated patients was small, complete agreement (100%) of the suspected cytology and malignant histology findings was shown only in category 5 of the EU-TIRADS classification (**Table 3**).

By increasing the number of examined subjects in future studies, it will be possible to define NOw levels by age and gender, increasing the positive predictive value of the procedure to define subjects requiring surgical treatment precisely. In addition, increasing the size of the examined population will also more clearly define the role of TGw, which according to our data, positively correlates with the cytology finding.

## 5 Concluding remarks

In this cross-sectional study, we reported that the levels of TGw positively correlate with the cytology finding of the biopsy in euthyroid subjects without levothyroxine therapy. A higher level of TGw correlates with higher categories of the Bethesda classification, i.e., it indicates a higher malignant potential. The levels of NOs, NOw, TGs, CTs, CTw, and the NOw/TGw ratio do not correlate with the cytology finding of the biopsy. The level of NOw positively correlates with the categories of the TIRADS classification. Higher levels of NOw correspond to the more suspicious US findings. Our study suggests that determining NOw, TGw, and NOw/TGw ratios has significant clinical importance in defining the nature of TN. Future population and prospective studies should investigate the importance of these measurements in the clinical setting to select patients for surgical treatment. The reason is that even a minimal decrease in the number of patients with benign TN who have been unnecessarily surgically treated and any increase in the detection of patients with malignant TN represent meaningful progress in daily thyroid clinical practice. Particularly intriguing is the evidence of dysregulation of microRNAs (miRNAs), long non-coding RNAs (lncRNAs), and circular RNAs (circRNAs) in certain thyroid neoplasms, as well as the potential application of these molecules as sensitive diagnostic and prognostic biomarkers. In line with that, the clinicians treating thyroid neoplasms would benefit significantly from the addition of such novel biomarkers.

## Data availability statement

The raw data supporting the conclusions of this article will be made available by the authors, without undue reservation.

## Ethics statement

The studies involving humans were approved by The Ethics Committee of University Clinical Hospital Zemun, Vukova 9, Zemun, Serbia (approval number 507/1). The studies were conducted in accordance with the local legislation and institutional requirements. The participants provided their written informed consent to participate in this study.

## Author contributions

Conception: VS and ZG. Performed the research: VS, ZG, MO, and SZ. Interpretation or analysis of data: VS, ZG, JG, MO, and SZ. Preparation of the manuscript: VS, ZG, MO, SZ, and MM. Revision for important intellectual content: EI, ME, XG, and MM. Supervision: EI. All authors contributed to the article and approved the submitted version.
